# A new anatomical approach of cervical lateral mass for cervical pedicle screw and paravertebral foramen screw insertion

**DOI:** 10.1371/journal.pone.0219119

**Published:** 2019-07-18

**Authors:** Moon-Kyu Kim, Ho-Jung Cho, Dai-Soon Kwak

**Affiliations:** 1 Department of Neurosurgery, Gangneung Asan Hospital, University of Ulsan College of Medicine, Gangneung, Republic of Korea; 2 Catholic Institute for Applied Anatomy / Department of Anatomy, College of Medicine, The Catholic University of Korea, Seoul, Republic of Korea; Virginia Tech, UNITED STATES

## Abstract

Thus far, anatomical studies have reported data on the cervical pedicle, with the focus remaining on the pedicle itself. It was necessary to obtain more comprehensive data about the relationships between the lateral mass, pedicle, and transverse foramen for cervical pedicle screwing (CPS) and paravertebral foramen screwing (PVFS), a new technique. The purpose of this study was to describe the relationships between the lateral mass, pedicle, and transverse foramen. This study analyzed computed tomography images from 77 patients (42 female, 35 male; mean age: 63.95 years). The anatomical pedicle transverse angle (PTA) and linear parameters of the lateral mass were measured, and the relationship between the calculated angles and the anatomical PTA was investigated. θp was defined as the convergence angle from the posterolateral edge of the lateral mass to the pedicle, and θc was defined as the convergence angle from the posterolateral edge of the lateral mass to the anterolateral corner of the vertebral foramen. The thickness of the cortical bone of the medial wall of the lateral mass (cT) and the medial (mT) and lateral (lT) walls of the pedicle at C3–7 were also measured. The PTA was similar to θp and θc at C3–6, but different at C7. In all cases, the transverse foramen was located more anterior to the posterior wall of the cervical body at C3–6, but not at C7. mT and cT were significantly thicker than lT at all levels. Lateral fluoroscopic images show that when the probe is inserted along θc, it meets the counter corner of the lateral mass at C3–6 without invasion of the transverse foramen if it does not cross the posterior wall of the vertebral body. This can be significant when performing CPS and PVFS.

## Introduction

Cervical pedicle screw insertion (CPS) has been gaining attention because CPS in the subaxial cervical vertebra provides biomechanical advantages in cases of cervical trauma and deformity; however, it is still considered a technically risky procedure owing to potential neurovascular complications [1, 2]. Thus, numerous applied anatomical studies for CPS have been conducted, focusing on the entry points of pedicle screws, transverse angles, or diameters of the cervical pedicle [3–9]. The anatomy of the cervical pedicle is important for CPS. However, in the real operative field, locating a pedicle behind the cervical lateral mass remains a challenge for surgeons; a review of studies about the CPS procedure consequently revealed an investigative emphasis on how to safely find the opening of the cervical pedicle behind the lateral mass [10–20]. Recently, paravertebral foramen screw fixation (PVFS) was introduced [21, 22]. This technique is safer than CPS, but it also demands understanding the anatomical relationship between the cervical lateral mass and its surrounding structures [22]. This means that the anatomical relationship between the lateral mass and the cervical pedicle is integral for CPS/PVFS; however, few previous anatomical studies have been conducted about the lateral mass. Moreover, those previous studies were limited to the general aspect of the lateral mass or the location of the transverse foramen without considering anatomical relationships between the pedicle, the transverse foramen and the lateral mass [3, 23–25]. Thus, a specific anatomical study of the lateral mass for CPS and PVFS is required. This study was conducted to determine detailed anatomical characteristics of the relationships between the lateral mass, transverse foramen, and cervical pedicle for CPS and PVFS. The objective of this study was to present anatomical measurements of the lateral mass and transverse foramen of the cervical vertebra, which may allow improved safety of CPS / PVFS procedures.

## Materials and methods

This study enrolled 77 patients (42 female, 35 male; mean age: 63.95 years) who underwent a cervical computed tomography examination at Gangneung Asan Hospital between December 2014 and February 2015. The CT image sets were collected retrospectively without prior consent under IRB approval (Gangneung Asan Hospital, No. 2014–55). Fully anonymized images (except for age and sex) were obtained from the department of radiology and used for analysis and measurement. None of the patients had any pathology in their cervical bone structures or severe degenerative changes of bone. The patients were scanned with a helical CT scanner (LightSpeed VCT 64; General Electric, Milwaukee, WI, USA; 0.625 mm slice thickness and the same reconstruction interval). A three-dimensional model was developed using the primary DICOM images, and axial images were re-formed parallel to the end plates of the vertebral body of each cervical segment by using Mimics 17.0 (Materialise Corp., Leuven, Belgium). The images of the pedicle and lateral mass at the largest pedicle diameter were chosen to measure the linear parameters, cortical bone thickness, and transverse angle of the pedicle.

**Fig 1:**
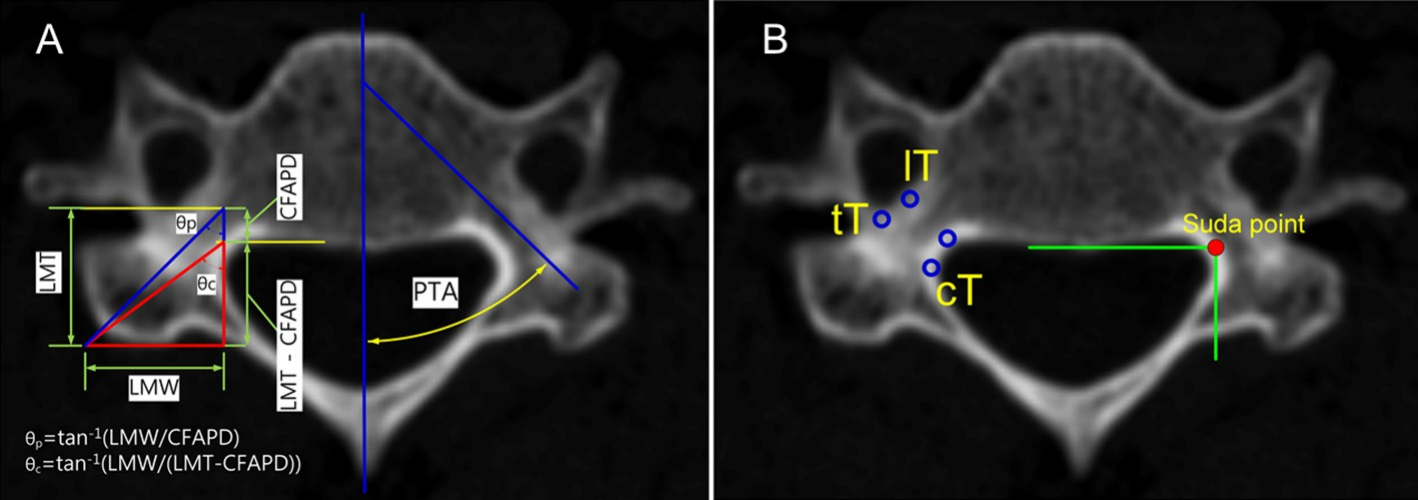
(A) Linear and angular parameters. PTA was real transverse convergence angle of the cervical pedicle. θp/θc represented calculated tangential angles based on linear parameters of the lateral mass. (B) Cortical bone thickness was measured at the four indicated points. mT; medial cortex of the pedicle, lT; lateral cortex of the pedicle, cT; medical cortex of the lateral mass (spinal canal), tT; posterior cortex of the transverse foramen.

The linear parameters of the lateral mass are shown in Fig 1A. Lateral mass parameters, including the abbreviations reported by Chazono et al., were used for comparisons [3]. The Lateral Mass Thickness (LMT) is defined as the distance from the posterior cortex of the transverse foramen to the posterior outer cortex of the lateral mass, and the spinal Canal to transverse Foramen Antero-Posterior Distance (CFAPD) is defined as the distance from the posterior wall of the vertebral body to the posterior cortex of the transverse foramen. The Lateral Mass Width (LMW) signifies the distance from the inner cortex of the most lateral vertebral foramen to the lateral outer cortex of the lateral mass. In this study, LMW was additionally measured to calculate the virtual angle from the posterolateral ridge of the lateral mass to the safe point (also called the Suda-point) and virtual opposite corner point (*θc* and *θp*, respectively; Fig 1A). The Pedicle Transverse Angle (PTA) is defined as the real transverse angle between the mid-sagittal plane of the vertebral body and the transverse pedicle axis.

Cortical bone thickness was measured at the medial and lateral cortex of the pedicle, the medical cortex of the lateral mass (the lateral cortex of the vertebral foramen), and the posterior cortex of the transverse foramen (Fig 1B). All measurements were collected by a single author. All of the landmark points for measurement were marked on images and confirmed by two other authors to minimize inter- and intra-observer error. Also, to determine the reliability of the measurements, 20 samples were selected randomly for repeated measurement by two authors. We assessed the statistical significance between the repeat measurement data, and the intra-observer error was evaluated by the intra-class correlation coefficient (ICC) value. All parameters were analyzed statistically (means and standard deviations), and differences of values were evaluated with a t-test and one-way analysis of variance (ANOVA).

## Results

The results of the reliability tests for inter-/intra-observer showed good agreement. The results from the repeated measurements showed no statistical difference in all measurement parameters (p<0.02), and the ICC values between the two observers ranged from 0.85 to 0.94. Table 1 displays the measurements of linear parameters (154 lateral mass and pedicles at each level, C3–7) along with relevant results from previous studies. The overall data obtained in this investigation were similar to those previously reported, and the differences in LMT and CFAPD could be attributed to differences in the measuring points. Our parameters were focused on the posterior cortex of the transverse foramen, and the thickness of cortical bone was excluded from the LMT and CFAPD values. LMT and LMW were largest in C5; however, there was no significant difference when compared to the values obtained in C3–7. The LMT of C7 was the smallest of all levels; however, there was no significant difference when compared to the measurements of other levels, and the calculated standard deviation was the largest. The number of occurrences of negative CFAPD values at each cervical level were obtained 6 (3.8%), 1 (0.6%), 10 (6.4%), and 15 (9.7%) times out of 154 measurements at C3–6, respectively. An intersex difference was noted in LMT and LMW (P < 0.01), but no significant difference was found in CFAPD.

**Table 1:** Linear parameters and results from previous studies. [unit: mm, mean±SD].

		C3	C4	C5	C6	C7
Stemper	LMW	11.1±1.3	11.4±1.2	12.4±1.2	12.8±1.4	11.8±1.2
Pait	LMW	11.47±1.99				
	LMT	15.04±1.98				
Miyazaki	LMW	12.1±2.0	12.1±1.6	12.9±2.1	12.3±1.5	12.1±1.3
	LMT	12.6±2.1	12.2±1.8	13.0±2.2	12.8±1.8	11.8±2.3
Chazono	LMT	12.2±1.6	11.8±1.5	12.3±1.4	12.6±1.6	10.7±2.0
	CFAPD	2.5±1.2	3.1±1.2	2.9±1.3	2.2±1.2	1.1±1.2
This study	LMW	12.23±1.11	12.45±1.17	12.78±1.47	12.50±1.50	11.88±1.83
	LMT	11.64±1.45	11.32±1.56	12.16±1.65	11.90±1.64	10.89±2.07
	CFAPD	1.58±1.13	2.26±1.22	1.85±1.41	0.96±1.50	-0.5±1.83

**Fig 2:**
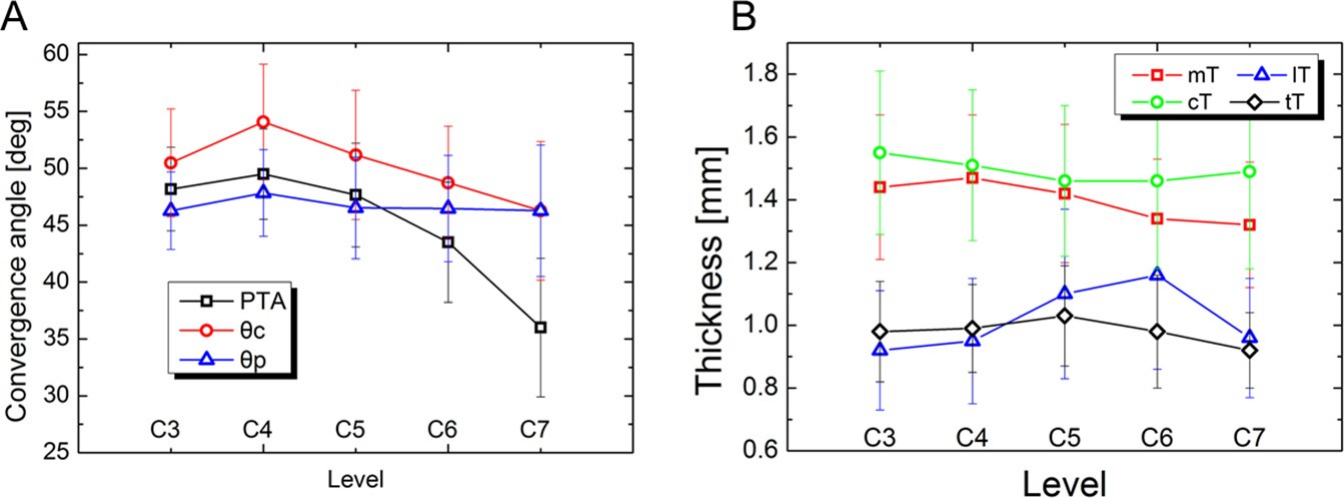
(A) PTA, *θ*p, and *θ*c had similar patterns except at C7. The angle difference was within 5° at C3–6. (B) mT/cT was significantly thicker than lT/tT at all levels. cT thickness remained constant thickness regardless of vertebral level.

Table 2 shows the angular parameters with relevant results from previous investigations. The PTA values measured in this study were similar to those of others. There were intersex differences in PTA at C3, C6, and C7 (PTAs were larger in males than in females; P < 0.01); however, there was no significant intersex difference at C4 and C5. The PTAs in males were 48.95° (SD 3.53), 44.68° (SD 5.60), and 37.51° (SD 6.20) at C3, C6, and C7, respectively, and 47.51° (SD 3.69), 42.48° (SD 4.78), and 34.76° (SD 5.74) at C3, C6, and C7, respectively, in females. The largest PTA was at C4. The calculated angles (*θc* and *θp*) were not closely correlated with the PTAs at each cervical level. However, the patterns observed in *θc* and *θp* were similar to those of PTA, and there were small differences of within 5° between the PTA and *θc* and *θp* at C3–6. On the other hand, these values were different at C7 (Fig 2A).

**Table 2:** Angular parameters and review of literature [unit: degree, mean±SD].

		C3	C4	C5	C6	C7
Panjabi	Rt	41.6±1.13	44.6±1.66	39.3±4.45	29.6±2.30	33.1±2.23
	Lt	42.9±2.16	43.9±2.47	41.2±4.77	34.1±2.16	26.7±2.69
Chazono		46.0±4.7	50.2±4.7	48.1±6.2	43.3±5.8	33.6±5.8
Reinhold		47.6±5.6	50.3±8.3	49.3±7.2	44.0±7.0	39.1±6.0
Chen		46.79±4.11	49±5.53	47.55±6.48	40.89±6.86	32.26±3.68
Munusamy	Chinese	44.6±4.78	48.2±4.63	47.3±4.04	43.8±4.65	37.7±5.09
	Malay	48.1±5.41	47.9±4.67	48.0±4.14	43.1±2.58	39.1±5.07
	Indian	51.2±5.21	53.4±3.42	53.6±1.96	48.3±3.18	41.3±6.40
This study	PTA	48.18±3.68	49.50±3.97	47.66±4.55	43.50±5.28	36.00±6.09
	θc	50.48±4.73	54.08±5.08	51.17±5.68	48.73±4.98	46.27±6.09
	θp	46.27±3.41	47.84±3.81	46.52±4.48	46.47±4.67	46.27±5.77

Table 3 shows the cortical thickness of the pedicle and lateral mass. The cortical bones at C3 and C4 were thicker in males than in females. The cortical thicknesses of mT, lT, cT, and tT were significantly different at the same level by ANOVA (cT > mT > lT > tT, P < 0.01).

**Table 3:** Cortical bone thickness at four points [unit: mm].

Level	Region	female		male		combined		p
		mean	SD	mean	SD	mean	SD	
C3	mT	1.38	0.22	1.51	0.22	1.44	0.23	**<0.01**
	lT	0.89	0.20	0.96	0.17	0.92	0.19	**0.01**
	cT	1.50	0.25	1.60	0.26	1.55	0.26	**0.01**
	tT	0.98	0.16	0.97	0.15	0.98	0.16	**0.68**
C4	mT	1.43	0.19	1.52	0.21	1.47	0.20	**<0.01**
	lT	0.91	0.19	1.00	0.20	0.95	0.20	**<0.01**
	cT	1.48	0.21	1.54	0.26	1.51	0.24	**0.01**
	tT	0.98	0.13	1.00	0.15	0.99	0.14	**0.17**
C5	mT	1.39	0.21	1.47	0.22	1.42	0.22	**0.02**
	lT	1.08	0.27	1.12	0.27	1.10	0.27	0.35
	cT	1.43	0.23	1.50	0.24	1.46	0.24	**0.04**
	tT	1.02	0.17	1.03	0.14	1.03	0.16	0.51
C6	mT	1.34	0.18	1.35	0.20	1.34	0.19	0.68
	lT	1.15	0.30	1.17	0.30	1.16	0.30	0.61
	cT	1.44	0.26	1.48	0.30	1.46	0.28	0.31
	tT	0.97	0.19	0.99	0.17	0.98	0.18	0.59
C7	mT	1.31	0.19	1.33	0.20	1.32	0.20	0.49
	lT	0.93	0.18	0.99	0.20	0.96	0.19	**0.05**
	cT	1.49	0.29	1.49	0.35	1.49	0.31	0.36
	tT	0.92	0.13	0.93	0.12	0.92	0.12	0.74

## Discussion

There are very few linear parameters of the cervical lateral mass, as shown in Table 1 (3, 23–25). Chazono et al. and Miyazaki et al. studied the linear parameters of the lateral mass for CPS, and their landmarks were different from the general anatomy in LMT; however, they did not assess the relationship between the lateral mass and pedicle (3, 23). The CFAPD data obtained in this study were different from those of Chazono et al. in terms of landmarks: our data excluded the cortical thickness of the transverse foramen that they included. Our CFAPD data demonstrate that the posterior cortical wall of the transverse foramen was located anterior to the posterior wall of the vertebral body at C3–6. A negative CFAPD signifies that the posterior wall of the transverse foramen was located more posterior than the posterior wall of the cervical body. While the occurrence rate of negative CFAPD was 0.6–9.7% at C3-6, the mean CFAPD at C7 was negative, indicating that the pedicle and lateral mass of C7 had characteristics similar to those of the thoracic vertebra. Negative CFAPDs obtained at C3-6 were very few and were mainly due to enlarged variation of the transverse foramen. Therefore, the results of this study showed constant relationships between the lateral mass and surrounding bony structures despite severe individual variation in linear and angular parameters.

**Fig 3:**
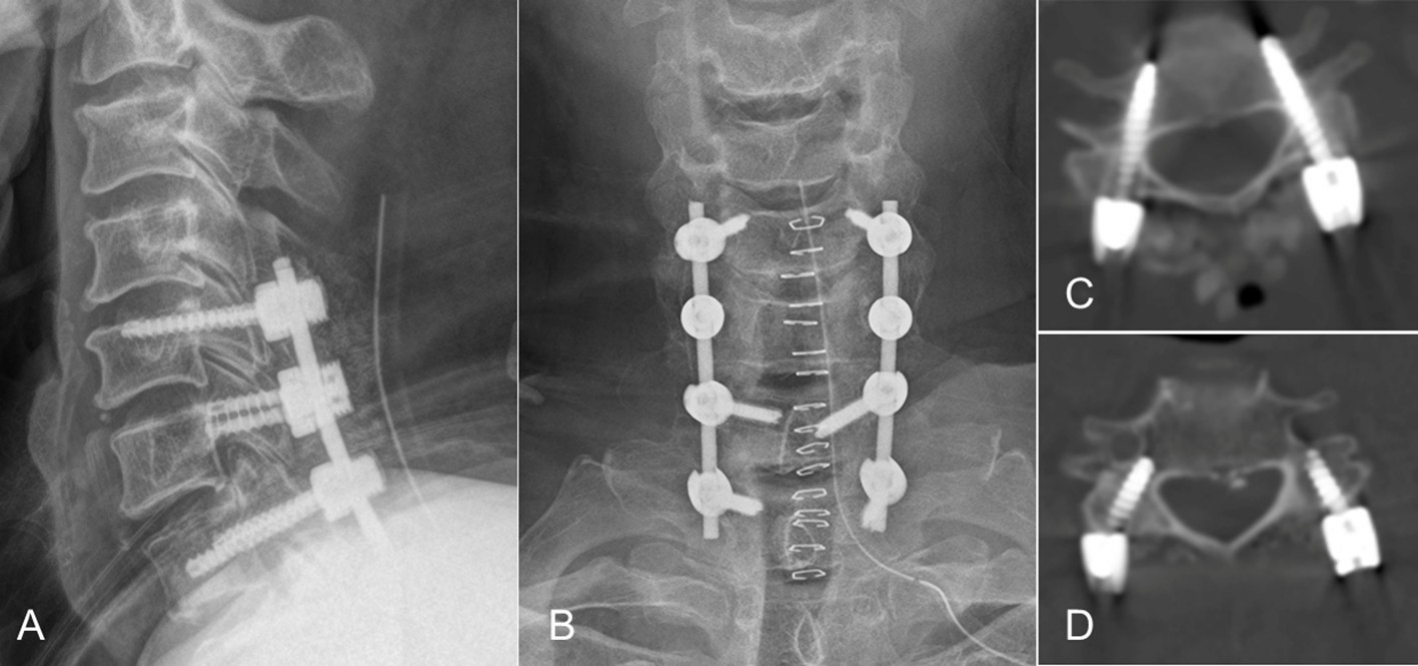
(A) (B) Postoperative radiography showing alternative usage of CPA and PVFS. CPS was employed at C5/7 and PVFS at C6. The depth of PVFS could be observed on a lateral image. The end point was just past the posterior wall of the body at C6. A-P image shows the adaptability of PVFS: no need of additional connecting rods. (C) Axial computer tomography image of C6. (D) PVFS at C6 was. The ideal location of PVFS was on the right. On the left side, PVFS was laterally located and touched the vertebral foramen but remained safe if the screw stopped just anterior of the posterior wall of the vertebral body.

**Fig 4:**
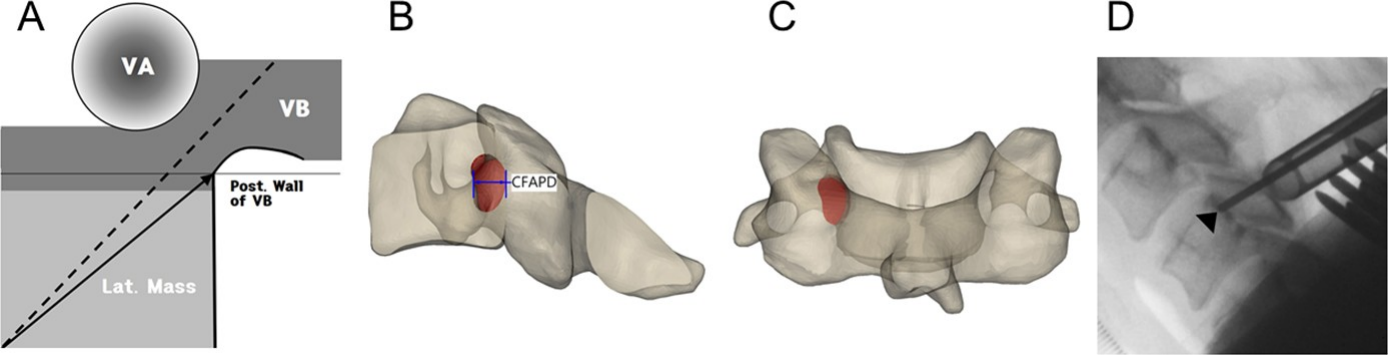
(A) Schematic drawing of C3–6 based on the results of this study. The thickness of the transverse foramen and lateral mass were similar to real cortical thickness, and the size of the lateral mass was similar to linear parameters. The dotted line represents PTA, and the solid arrow demonstrates the diagonal path introduced by θc. The tip of the arrow indicates the Suda-point. (B) 3D model sagittal projection image with the entry of the pedicle, CFAPD means safe space for searching the entry of the pedicle on the lateral view. (C) coronal projection image of the 3D model. (D) Lateral image showing that the tip of the probe met the posterior line of the vertebral body at the Suda-point.

Subaxial CPS is an effective procedure for treating trauma, deformity, and other diseases because of its mechanical superiority over other fixation methods [1, 2, 26, 27]. However, it bears the potential risk of causing neurovascular complications originating from the anatomical environment, as observed in a remarkable number of anatomical studies [3–7, 9]. Panjabi and colleagues evaluated the internal morphology of the cervical pedicle and determined that the medial wall of the pedicle is much thicker than the lateral wall [28]. These anatomical information details provided some guidance for CPS, and numerous reports for CPS techniques suggested that the medial wall of the pedicle is a safe marker for entry to the cervical pedicle [10, 12, 13, 16–19, 26, 29]. However, these technical notes lacked some anatomical data because they also used the medial wall of the lateral mass as a marker prior to entering the cervical pedicle. They did not explain why contact of the medial wall of the lateral mass is important for finding the opening of the pedicle. Recently, only one study evaluated the strength of the medial cortex of the lateral mass [30]. Studies on the complications of CPS provided further indications on the proper screwing of the cervical pedicle; that is, major misplacements of CPS involving the transverse foramen occurred in the lateral part of the pedicle, but involvement of medial wall was relatively rare. Spinal cord injury by CPS misplacement was also very rare [31, 32]. While these findings offered indirect pointers for CPS techniques, it was still necessary to determine the thickness of the medial wall of the cervical lateral mass. Information about the thickness of the medial wall of the cervical pedicle was only useful after passing the opening of the pedicle. In our study, we found that the medial wall of the cervical lateral mass was significantly thicker than the wall of the transverse foramen (Table 3). This can be basic data used to support previous technical reports in which the medial wall of the cervical lateral wall was used as a guide. There have been no previous reports on the thickness of the medial wall of the cervical lateral mass, even though it can be easily demonstrated during routine computed tomography. From a scientific point of view, it is also necessary to define the medial wall of the cervical lateral mass and confirm that it is as thick as the medial wall of the pedicle. The medial wall of the lateral mass originates from the same part of the neural arch as that of the pedicle, like the ventral cortical bone of the lamina in the thoracic level [33]. Cortical thickness assessments showed that cT was the thickest region and was similar to mT. Fig 2B shows the pattern of thickness at each site of measurement. Based on previous data and the results of this study, the lateral mass is observed as a rectangular shape on the axial computed tomography image (parallel plane to end-plate of the vertebral body), and the pedicle is attached to the anterior medial corner. The CPS starting point is near the posterior lateral corner. A probe should be introduced diagonally to the counter corner to locate the pedicle easily. The diagonal line to the counter corner of the pedicle (introduced by the angle of *θp*) had a very similar pattern and value to that of the PTA at C3–6, and the diagonal line to the Suda-point (introduced by the angle of *θc*, Figs 3D and 4A) showed the same pattern within 5° at C3–6, although its values were different from those of PTA than in *θp* (Fig 2A). This means that the probe touches the Suda-point safely if it is introduced using angle *θc*, and that the probe can be adjusted into the opening of the pedicle by touching the medial wall of the lateral mass because PTA is within 5° of *θc*. For this reason, some surgeons use a straight, stiff probe. It is very difficult to adjust the probe if PTA is different from *θ*c. However, this result could not be used as a reference for screwing at C7 owing to the thin and irregular shape of the lateral mass and the more posterior location of the transverse foramen.

PVFS was introduced as an alternative procedure for posterior cervical fixation and is theoretically safer than CPS on account of stiffer lateral mass screw fixation [22]. The entry points include the middle or more lateral points of the lateral mass. It was quite difficult to change to another technique when conducting either CPS or lateral mass screw fixation. The difference of entry points in these two techniques made loading a rod to the screw heads challenging; thus, an additional connecting rod was sometimes required. The adaptability of entry points in PVFS can therefore be useful for salvage screw fixation (Fig 3B); however, a safe depth limit was not established. Our CFAPD data suggest a safe depth for PVFS, namely, that PVFS can be safe at C3-6 if the screw was inserted up to the line of the posterior wall of the cervical vertebra as observed on lateral fluoroscopic images and up to 1-2mm more anteriorly after passing the line of the posterior wall of the cervical body at C3-6 (Figs 3A, 3D and 4B).

CFAPD data, angles, and cortical thickness are schematically illustrated in Fig 4A and provide a clinical strategy for CPS. If a pedicle probe is inserted around the posterior lateral corner at about 45–50° (depending on the starting point), the probe reaches the counter corner (the Suda-point) safely. The probe will be close to the posterior line of the vertebral body, as observed under lateral fluoroscopic image guidance (Fig 4B). If the probe is located posterior to the posterior line of the vertebral body, it can be adjusted anteriorly by “feeling” the thick medial cortical wall of the lateral mass. When the tip of the probe reaches the Suda-point, the probe can be marginally adjusted by slightly changing the angle to within 5° and introducing it into the cervical pedicle.

Understanding the constant relationships between the cervical lateral mass and connecting structures could serve as anatomical evidence for techniques of CPS insertion and as a safe depth guide for PVFS insertion.

## Conclusions

There are currently various CPS techniques; however, these techniques are generally based on complete comprehension of the lateral mass and cervical pedicle. Even if surgeons already know of these features, the anatomical data presented in this study could be helpful as evidence for existing techniques and as guiding points for developing new CPS and PVFS techniques.
